# The Importance of the Inter-Articular Fibro-Cartilage of the Temporo-Mandibular Articulation

**Published:** 1918-07

**Authors:** Richard Summa


### THE IMPORTANCE OF THE INTER-ARTICULAR FIBRO-CARTILAGE OF THE TEMPORO-MANDIBULAR ARTICULATION

BY RICHARD SUMMA, D.D.S.

Every textbook on dental prosthetics devotes a chapter to the anatomy of the temporo-mandibular articulation, and many laudable efforts to devise a method of reproducing the condyle path upon dental articulators are recorded. Only the osseous structures of this articulation however, have been given any practical consideration in the attempts to construct a mechanical apparatus which would imitate the complex mandibular movements. The soft tissues, of which the inter-articular fibro-cartilage is the most important, have been almost entirely disregarded. It is true that all textbooks mention and vaguely describe this meniscus, nevertheless one is reminded of the mention which occlusion of the teeth was given in treatises on orthodontia prior to the days of Dr. E. H. Angle's recognition and application of this anatomical factor as the basis of all orthodontic procedures.

Unfortunately, like other parts of the human anatomy which are of primary and almost exclusive interest to the dentist, the meniscus has been given honorable mention by anatomists in the description of the temporo-mandibular articulation, and there the matter rested. It is quite in keeping with the natural order of things that each specialty should seek and develop the knowledge pertinent to its own progress, and thereby strengthen itself. It is needless to state that the prosthetist's and the orthodontist's chances for studying this articular fibro-cartilage *in situ* are few and far between. It becomes necessary not only to obtain access to an anatomical laboratory, but also to elicit the assistance of an anatomist, and hence I believe the efforts of Dr. Henry J. Prentiss, professor of anatomy at the State University of Iowa, will readily meet the appreciation of the dental profession.*

In a recent discussion relative to mandibular movements the disputants agreed that the temporo-mandibular articulation (and this must include all the structures concerned in this articulation) is originally shaped in response to the occlusion of the teeth. It was likewise agreed that malocclusion and loss of teeth produce corresponding changes in the temporo-mandibular articulation.

The investigation of Dr. H. J. Prentiss confirms these assumptions; furthermore, it calls attention to the fact that the meniscus is not the least important component of the temporo-mandibular articulation. The meniscus is that component part of this articulation which first receives the stimulus and also sustains the first impact to this joint resulting from the function of mastication.

Dissections of this joint clearly reveal the fact that following the loss of a few teeth the meniscus becomes thinned; following the loss of a larger number of teeth the meniscus is perforated, while following the loss of all teeth the entire upper surface of the condyle becomes denuded of inter-articular fibro-cartilage. Immediately following the perforation of the meniscus, absorption of the correspondingly exposed surfaces of the condyle and the glenoid fossa begins, and thus the change and reshaping of the temporo-mandibular articulation take place. It may not be amiss in passing to reiterate and emphasize the observation in the material thus far examined that the perforation of the meniscus always begins in the same location, namely, the external lateral angle.

From all that can be gleaned from the writings and discussions of the subject, the prosthetist and the orthodontist happily assume that this joint will readapt itself

---

* See paper by Dr. Prentiss entitled "A Preliminary Report upon the Temporo-Mandibular Articulation in the Human Type," at page 505 of the June issue of the *Dental Cosmos.*

to the occlusion which their handicraft sees fit to establish—the one with artificial dentures and bridge work, the other with a corrected or improved occlusion of the natural teeth. However, practitioners of these branches of dentistry have frequently had occasion to wonder why such blissful dreams rarely come true. After enjoying the privilege of studying the anatomical material from which Dr. Prentiss derived his conclusions, one should be pardoned for wondering why the prosthetist's devices ever give reasonable satisfaction in any instance. At the same time one can not help but give renewed expression of thankfulness to tolerant mother Nature for granting as many reasonably good results to orthodontic operations which involve a change in the temporo-mandibular articulation.

The prosthetist's efforts are expended on individuals of mature years when destructive processes of osseous structures are more the order of the day than constructive growth. If cognizance is also given to the fact that a non-vascular organ, such as this inter-articular fibrocartilage, has no recuperative or reconstructive power, then we can be little short of amazed by the assertion that it is the prosthetist's privilege and duty to establish an articulation of artificial dentures irrespective of the temporo-mandibular articulation of any given case, because the occlusion of the teeth of artificial dentures decreed by the prosthetist will induce the matured structures of the temporo-mandibular articulation to grow as he wishes them to grow. The difficulty with this class of prosthetists has been that they based their arguments upon what they could do, because that is plainly visible, giving no thought to conditions encountered in the living subject which are not necessarily amenable to their mechanical efforts.

The prosthetist is at a disadvantage because his restorations must be adapted to abnormal condyle paths of infinite variations. In the largest proportion of cases of artificial dentures and bridges we must expect to deal with thinned or perforated menisci. It is a universal experience that artificial dentures will permit of exerting only a small fraction of the force capable of being exerted by natural teeth. This has been attributed solely to the mobility of the bases upon which artificial dentures must necessarily be constructed. While this is unquestionably an important factor, may we not assume that the loss of power and masticating efficiency is also in part due to an instinctive hesitation on the part of the patient to exert more force, to avoid at least a feeling of discomfort, not to say pain? Occasionally, patients for whom apparently well-occluding bridges have been made will complain of a loss of masticating power or of an interference with the freedom of mandibular movements, or according to a recently published article, that the bridges "feel like stone walls." These complaints have been ascribed to a neurotic tendency in the patient, or to excessive peevishness, etc. However, such conditions are more correctly explained by the knowledge that in such cases the normally intervening fibro-cartilage has been irreparably destroyed, and as a result the denuded bone surfaces of this articulation have been forced into frictional contact by this so-called improved occlusion.

There are types of malocclusion which present a short horizontal ramus or body of the mandible, usually characterized by a receding chin. These cases suggest the necessity of carrying the mandible forward by means of intermaxillary force or by means of a bite plane, and the operator has proceeded to "jump the bite" in the endeavor to produce a change in the temporo-mandibular articulation which would permanently compensate for the shortness of the horizontal ramus.

Practical experience in such cases proves the impossibility of achieving the desired results, and a study of the anatomy of the articulation stamps such an orthodontic operation as questionable. Whatever improvement is possible in these cases is due to a correction of the occlusion of the teeth and the consequent molding of the body of the bone.

It is immaterial whether or not, at this time, the difficult task of constructing a device upon which the mandibular movements can be imitated has been accomplished. Nevertheless, a sincere appreciation is due the men who have intuitively or otherwise recognized the futility of placing the cart before the horse. Most deserving of mention in this connection is the work of Dr. Gysi.

The argument that some of the dentures constructed according to any of the methods which ignore existing natural conditions are successfully worn, or that fixed bridge work which has violated the important demand for mobility of the teeth has been paid for and worn by that same patient individual, reminds one of the argument that individuals whose wounds have been maltreated and have survived in the struggle for existence. If scientific men were content to depend upon such reasoning it would indeed be deplorable.—*Dental Cosmos.*

